# The Effect of Psychological State on Pain Perception in a Simulated Dental Setting Among Young Adults: A Randomized Comparative Experiment

**DOI:** 10.3390/dj14070431

**Published:** 2026-07-13

**Authors:** Sonja Derouiche, Per M. Aslaksen, Jan-Are K. Johnsen

**Affiliations:** 1 Department of Clinical Dentistry, Faculty of Health Sciences, UiT The Arctic University of Norway, 9037 Tromsø, Norway; 2 Department of Psychology, Faculty of Health Sciences, UiT The Arctic University of Norway, 9037 Tromsø, Norway; per.aslaksen@uit.no

**Keywords:** pain perception, psychological state, emotion, attention, stress

## Abstract

**Background/Objectives**: The psychological state affects perceived pain and can be modulated by emotions and attention. Pain experiences are prevalent in dental treatment and play a key role in the development of dental anxiety and treatment avoidance. This study hypothesizes that video and audio that are either pain-relevant or neutral will impact pain perception, self-reported stress, and physiological stress in a dental setting differently. **Methods**: Eighty participants were randomized into a no-stimuli control group and four experimental groups, in which they were exposed to pain-relevant or neutral video or audio recordings. Heat pain was administered to the participants’ forearms before and after the video or audio exposure, and participants rated the pain using a computerized visual analogue scale. Skin conductance levels (SCLs) were measured to indicate arousal levels. **Results**: Pain reports were significantly higher in the control group compared to all other groups (all *p*-values < 0.05), except for those who viewed the surgical video. Also, both pain reports and SCLs were significantly higher during the pain-relevant video compared to the neutral video (*p* < 0.05). **Conclusions**: The interplay between the psychological state, emotion, attention and pain perception in dental care should be explored further, particularly regarding its potential relevance for pain control and supportive clinical environments.

## 1. Introduction

Pain is best understood as a biopsychosocial phenomenon and a complex experience shaped by biological factors related to physical sensation, psychological factors such as emotions and thoughts, and social factors including environment, relationships and culture. This perspective recognizes that pain has both sensory and affective components [1,2].

Pain is prevalent during dental treatment, and some studies estimate that 20–30% of patients experience moderate to severe pain during dental visits [3,4]. Despite advances in anesthetic techniques, a substantial proportion of patients still report pain during dental procedures [5,6]. For instance, studies of orthodontic treatment have found that approximately 90–95% of patients report experiencing pain during orthodontic treatment procedures [7,8]. In light of this, understanding more about what factors influence the experience of pain in the dental context becomes important.

It has been documented that psychological factors, such as attention and emotion, have an important influence on pain perception [9]. Attention is the most studied psychological factor that modifies the pain experience. Clinical and experimental studies show that pain is less intense when people are distracted [10,11], and when attention is withdrawn from noxious stimuli, reported pain decreases. For example, pediatric dental patients distracted with virtual reality (VR) report significantly lower pain during short invasive procedures than those who are not distracted [12]. Neuroimaging supports this, as studies utilizing functional magnetic resonance imaging (fMRI) and positron emission tomography (PET) show that distraction reduces activity in brain regions processing both the physical aspects of pain (how strong and where it is, the primary and secondary somatosensory cortex) and the emotional aspects of pain (anterior cingulate cortex and the insula) [10].

Furthermore, emotional state shapes pain perception, where negative emotions increase pain, while positive emotions reduce it. Anxiety levels appear to increase the experience of pain [13]. It has been found that mood influences pain in chronic illness, for example cardiac patients with depression report greater pain than non-depressed patients [14]. Also, experimental studies have found that mood-enhancing stimuli, such as calming scents, reduce anxiety [15,16].

Pain perception is susceptible to priming [17]. Priming refers to the phenomenon whereby prior exposure to a stimulus (e.g., words, images, sounds or contextual cues) influences the processing of subsequent stimuli, often without conscious awareness. Evidence suggests that pain perception is strongly shaped by contextual, social and environmental cues rather than nociceptive input alone. Experimental studies show that negative and pain-relevant verbal primes increase perceived pain intensity, especially at short stimulus intervals and high pain intensities, supporting the affective modulation of pain through motivational priming [18]. Complementing this, social cue studies demonstrate vicarious facilitation of pain, where observing others’ pain expressions enhances subsequent pain ratings and facial pain responses, with evidence for both affective and motor priming mechanisms [19]. In regard to the neural foundations of priming, recent neuroimaging findings indicate multimodal “vicarious body maps” linking visual and somatotopic representations, providing a possible brain mechanism for how observed bodily states influence one’s own sensory processing [20]. Finally, a systematic review of environmental interventions (e.g., calming music, virtual reality scenarios, lighting conditions) shows that structured sensory environments can often reduce pain, highlighting the clinical relevance of contextual modulation for pain management [21].

In dental settings, patients’ fear is often triggered by specific stimuli, including particular instruments, smells and sounds. A study indicates that invasive procedures (e.g., surgical tooth extractions) are among the most anxiety-provoking stimuli of dental treatment [22]. Because dental settings are rich in contextual cues, it is important to understand how they influence patients’ pain. In patients with anxiety, fear and phobia heightened electrodermal activity (EDA) has been observed when using various provocation methods, such as fear imagery or phobic pictures [23,24]. Elevated autonomic reactivity has also been linked to dental phobia when elicited by phobia-related images or video material [25,26]. Compared with static pictures, videos provide more realistic scenarios of the feared situation. Overall, EDA is a reliable indicator of sympathetic nervous system activation associated with autonomic arousal in both healthy and clinical samples [27].

Video and audio materials with negative and neutral emotional content can influence pain perception and autonomic arousal. Prior work investigating video-induced mood effects supports the use of video stimuli in pain research [28], and videos have reliably induced emotional states in participants [29,30]. Although both auditory and visual stimuli convey emotional cues, multimodal stimuli, combining both visual and auditory inputs, should be more effective than single-modality stimuli in inducing emotions.

Patients undergoing the same dental procedure can report noticeably different pain experiences. These differences matter clinically because adverse experiences can lead to the development of dental anxiety or dental phobia [31], which often leads to avoidance of dental care [32,33,34] and greater reliance on sedation [35]. Yet, few studies have examined how multimodal emotional priming, combining visual and auditory stimuli, influences pain perception and psychophysiological responses in dental settings.

The aim of this study was to examine whether pain-relevant video and audio stimuli influence pain ratings, subjective stress and skin conductance levels in a simulated dental setting compared with neutral stimuli and a no-stimulus control condition. We hypothesized that pain-relevant audio and video stimuli would increase reported pain and stress, both self-reported and measured by SCLs, relative to neutral stimuli in a simulated dental setting.

## 2. Materials and Methods

Eighty (80) healthy young adults aged 19–31 years were recruited via campus posters at UiT the Arctic University of Norway from September–December 2016. They were informed about the aims of the study and notified that participation was voluntary. All participants provided written informed consent and indicated that they were in self-reported good general health. Exclusion criteria were any diagnosed heart failure or arrhythmia, renal disease, cancer, epilepsy, chronic pain conditions, depression, bipolar disorder, schizophrenia or disabling developmental disorders. Participants received a gift card worth 300 NOK for the reimbursement of expenses. The study was approved by the Regional Committee for Medical and Health Research Ethics (Ref no. 2016/440). This study was not prospectively registered in a clinical trial registry.

### 2.1. Stimulus Material

Video and audio materials: All stimulus materials were recorded in 720p HD using a Zoom Q2HD camera (Zoom Corporation, Tokyo, Japan). The neutral video presented a third-person view of a dental hygiene procedure (tooth brushing, flossing, rinsing). The negative valence video presented a third-person view of an invasive surgical tooth extraction (incision, flap elevation, osteotomy, luxation and elevation). Videos were edited to a 5 min duration in iMovie version 10.1 on a MacBook Pro computer (Apple, Cupertino, CA, USA). Soundman OKM–II binaural in-ear microphones (Soundman e.K, Eberswalde, Germany) were used during the video recordings to provide a realistic three-dimensional soundscape for both scenarios [36]. The audio recordings from the videos were extracted using Audacity software version 2.1.2 [37] to be used as stimuli in the audio-only condition.

Pain stimuli: Pain was induced by a Pathway aluminum 30 × 30 mm contact heat thermode (Medoc Ltd., Ramat Yishai, Israel) placed on the glabrous skin of the right forearm of the participants. The thermode had a baseline temperature of 32 °C when applied to the arm. The duration of the pain stimuli was 15 s with a plateau for approximately 10 s at the target temperature of 47 °C. The temperature of 47 °C was predefined because it is a temperature that most people find painful [38].

### 2.2. Measures

Pain experience and subjective stress: Pain was measured continuously with a computerized visual analogue scale (CoVAS). During each heat stimulus, participants moved a slider along a 0–100 scale to indicate pain intensity (0 = no pain, 100 = most intense pain imaginable; [39]). Subjective stress was assessed with two adjective pairs from the Norwegian translation of the Short Adjective Check List (SACL), tensed/relaxed and nervous/calm rated on 0–10 scale [40].

**Figure 1:**
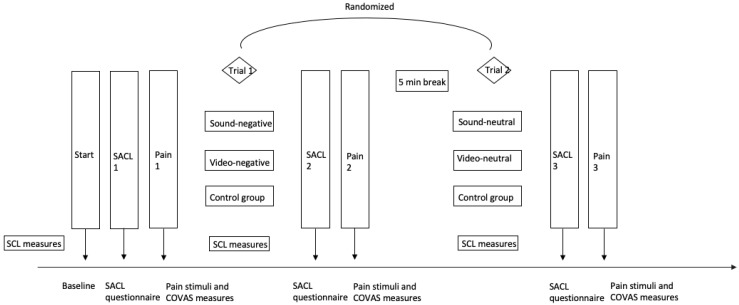
Overview of the experimental procedure. Participants first completed baseline SACL questionnaire and baseline pain assessment using contact heat stimulation and CoVAS ratings. Participants were then exposed to either video, audio or no stimulus according to group allocation. After each exposure period, SACL ratings and pain assessments were repeated. In the video and audio groups participants completed two trials with opposite stimulus valence, neutral and pain-relevant, separated by a 5 min break. The control group received no external stimulus during both exposure periods. SCL was recorded continuously throughout baseline and exposure periods.

Electrodermal activity (EDA): EDA measures continuous fluctuations in skin conductance driven by sympathetic sweat gland activity and is widely used as an indicator of psychological and physiological arousal [27]. In this experiment, we analyzed the tonic component, skin conductance level (SCL, in microsiemens µS), as an index of autonomic arousal. EDA was recorded with a Biopac MP150 using the BioNomadix Wireless EDA amplifier (Biopac Systems Inc., Goleta, CA, USA). Two EL507 electrodes were attached to the phalanges of the first and second fingers of the left hand. Signals were recorded at 2000 Hz with the Biopac AcqKnowledge version 4.4 [41] software and processed offline. Mean SCL was calculated for the baseline and for the two 5 min periods during which participants viewed video, listened to audio, or received no stimulus (see Figure 1).

Dental trait anxiety: Dental anxiety was assessed with the Modified Dental Anxiety Scale (MDAS; [42]). Participants rated fear in five imagined dental situations on a 1–5 scale, item scores were summed to yield a total score of 5–25 (higher score = greater anxiety). Following common recommendations, a score > 19 was used as the clinical cutoff for dental phobia [43].

### 2.3. Design and Procedure

Participants were randomly assigned by drawing one of five pieces of paper from a box upon entering the laboratory. Each piece of paper showed a number between 1 and 5, which assigned the participant to one of the experimental groups/conditions. The process was adaptive so that groups that had received the estimated number of participants determined necessary by the power calculations were removed from selection. The participants were assigned to a repeated mixed factorial design featuring a within-subjects valence factor (negative/pain-relevant vs. neutral) and a between-groups modality factor (video vs. audio), plus a control group receiving no stimuli. This yielded five conditions: video-neutral, video-negative, audio-neutral, audio-negative and control. Blinding of participants and investigators was not feasible due to the nature of the experimental stimuli. Participants were exposed to video, audio, or no stimulus and the investigators administering the procedure were aware of the group allocation.

Upon arrival at the Department of Clinical Dentistry laboratory, configured as a dental office with a functioning unit, participants were greeted by a female experimenter. After receiving study information and providing written informed consent, participants completed the MDAS and were seated in the dental chair facing an LCD screen. A contact-heat thermode was attached to the glabrous skin of the right forearm, and two electrodes (EL507) were placed on the first and second fingers of the left hand.

The session began with a 5 min SCL baseline (quiet rest), followed by the SACL. A baseline pain block was then delivered, consisting of two 15 s stimuli (target 47 °C) separated by 60 s. Then, the participants were provided with noise-cancelling headphones and completed Trial 1 according to group assignment: 5 min of video, 5 min audio, or 5 min sitting quietly (control). SCL was recorded continuously during the 5 min period. Immediately afterward, the SACL was repeated, the thermode site was shifted by one thermode length to minimize skin adaptation, a post-trial pain block (two 15 s stim, 60 s pause) was administered.

After another thermode site shift and 5 min break. Trial 2 was conducted with the opposite valence to Trial 1 (neural/pain-relevant) for the video/audio groups; the control group again sat quietly. The SACL was obtained a final time, followed by the last pain block. See Figure 1 for an overview of the procedure.

### 2.4. Sample Size Calculations

The sample size was calculated a priori using the software G*Power version 3.1.9.7 [44]. For a repeated-measures design with within- and between-groups interactions, a total of 70 participants were required to detect significant between-group effects, assuming small effect sizes (η^2^ = 0.01, *d* = 0.2). The analyses were performed with linear mixed models, but the sample size estimation is comparable to repeated-measures analysis of variance because of similar covariance matrix assumptions [45]. The sample size calculation was based on 3 groups, 5 conditions, 3 repeated measurements, power (1 − β) = 0.8, correlation between repeated measures = 0.5, nonsphericity correction (ε) = 1, and *p* = 0.05. The critical F value for this calculation was 2.01.

### 2.5. Statistical Analyses

The distributions of data for pain, SCL and stress were not significantly different from the normal distributions shown by the Shapiro–Wilk test, and this was further confirmed by an inspection of the Q-Q and box plots of the residuals. Linear mixed models (LMM) were chosen over standard linear models (e.g., repeated measures analysis of variance) because this method is suitable for analyzing data with unequal group sizes, handles missing data without losing power in the analyses compared to standard general linear models, and allows for combinations of both fixed and random effects [46]. An autoregressive covariance structure of the data (AR1) was found to produce the best fit to the data in all LMMs, according to the Akaikes information criterion and the −2 log likelihood parameter. The participants were assumed to induce significant individual variance, and the individual variance was treated as the only random effect in the repeated measures analysis. The group was entered as the only fixed factor in the analysis, whereas sex, age and MDAS scores were covariates. Only the main effects of the included variables were tested due to the small group sizes. The *p*-values for pairwise comparisons between the groups were adjusted for multiple comparisons with Bonferroni corrections. Accordingly, the reported *p*-values for group comparisons are the adjusted values. *p*-values < 0.05 were considered significant. Analyses were conducted in SPSS versions 25 and 31 [47].

## 3. Results

### 3.1. Descriptive Statistics

Of the 80 participants, 50 (62.5%) were female and 30 (37.5%) were male. The mean age was 22.7 years (SD 2.65, range 19–31). The mean MDAS score was 8.55 (range 5–18). No participants scored above the suggested cutoff for dental phobia (MDAS > 19), although five participants met the threshold for substantial dental fear (MDAS ≥ 13). No sex differences were observed in MDAS scores. See Table 1 for descriptive statistics.

### 3.2. Pain Reports and Subjective Stress

**Figure 2:**
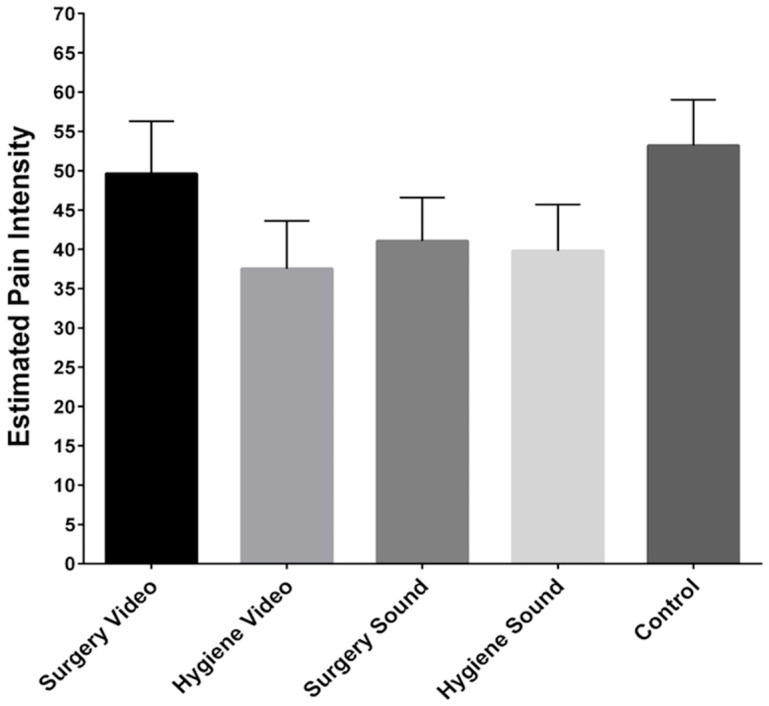
Estimated pain intensity by condition. Mean CoVAS pain ratings (0–100) for surgery video, hygiene video, surgery audio, hygiene audio and control (no stimulus).

Figure 2 shows mean pain ratings for the control and the four experimental conditions. A linear mixed-model analysis of pain reports was carried out, where the group was entered as the only fixed factor and sex, age, and MDAS scores were the covariates. Three participants were excluded from the analyses due to missing pain data. The effect of the group was significant; F (4,117) = 3.37, *p* < 0.05, η^2^ = 0.10. Pain reports were significantly higher in the control group compared to all other groups (all *p*-values < 0.05, Bonferroni-corrected), except for the comparison between the control group and those who viewed the surgical video (*p* = 0.57). Also, pain reports were significantly higher during the pain-relevant video compared to the neutral video (*p* < 0.05). No other group comparisons were significant after the Bonferroni corrections of the pain data. The female participants reported higher pain levels compared to male participants; F (1,73.5) = 4.32, *p* < 0.05, η^2^ = 0.06. No significant effects were found related to dental anxiety or age of the participants. The pain reports were significantly affected by individual variance, as shown by the random effect parameter (variance = 574.28, 95% CI (398.98–826.59), Wald Z = 5.38, *p* < 0.001).

Regarding subjective stress (SACL), the group effect was not significant, and no between-group comparison reached significance. Also, no sex differences were found. Younger age was associated with higher stress; F (1,381.22) = 15.56, *p* < 0.001, η^2^ = 0.04; and higher MDAS scores predicted higher stress; F (1,381.27) = 46.22, *p* < 0.001, η^2^ = 0.11. The stress reports were significantly influenced by individual variance, as shown by the random effect parameter (variance = 1.10, 95% CI (0.88–1.36), Wald Z = 9.08, *p* < 0.001).

### 3.3. SCL/Autonomic Measures

The group effect was significant on the measurements of SCL; F (6,101) = 2.45, *p* < 0.05, η^2^ = 0.13. The SCLs were significantly higher during the pain-relevant video compared to the neutral video (*p* < 0.05). No other group comparisons were significant after the Bonferroni corrections in the EDA data. The SCL measures were influenced by age, with lower age being associated with an increase in SCLs; F (1,74) = 4.22, *p* < 0.05, η^2^ = 0.04. No significant effect of sex was observed. The SCLs were significantly affected by individual variance, as shown by the random effect parameter (variance = 3.91, 95% CI (2.69–6.67), Wald Z = 5.26, *p* < 0.001).

## 4. Discussion

The primary aim of this study was to test whether audiovisual stimuli that were relevant or irrelevant to pain would alter pain perception, as well as psychological and physiological stress in a dental setting. Findings provide partial support for our hypotheses. First, higher pain ratings and SCLs were reported for the pain-relevant video compared to the non-pain-relevant video, but not for the other conditions. The reduction in pain ratings observed in the neutral video condition may reflect distraction rather than specific emotional priming. Second, and interestingly, the participants in the control group (no stimuli) reported higher pain than all experimental groups except the pain-relevant video group. In the absence of external stimuli, participants in the control group may have directed maximum attentional resources toward the upcoming nociceptive input. This may have increased somatosensory hypervigilance [48], which could contribute to higher pain ratings compared with the stimulus conditions. We would argue that the paradoxical effect related to the control condition is related to the attentional effects of attending to no stimuli versus attending to something specific, where the latter confers a clear distraction benefit. These findings align with extensive evidence that pain perception and physiological arousal are influenced by psychological processes [49]. Emotional valence modulates pain in opposite directions; negative emotions tend to increase pain, whereas positive emotions tend to decrease it [50], which underscores that pain is not driven solely by nociceptive input [51]. The absence of strong between-condition differences in our study might point to the emotional valence values of the stimuli being too similar. Possibly, the inclusion of a positive-valence condition (instead of a neutral condition) could have made the potential differences clearer.

Further, both priming and attention play a key role in pain modulation. The priming effect has an impact where pain-related cues (e.g., the dental unit, instruments, drilling sounds and invasive images) prepare the participant/patient to expect pain. Attention also plays a key role in pain modulation [52], and this is also supported by neuroimaging studies [53,54]. In the present study, both video and audio stimuli produced a distraction effect on pain. The control group (no stimulus) reported higher pain than the stimulus groups. This effect was strongest for neutral, multimodal content (hygiene video), whereas negative procedure-related content (surgery video) showed no significant distraction effect. Overall, pain decreased when attention was directed away from the nociceptive input, but the distraction benefit was attenuated when the stimuli were closely tied to painful dental procedures.

From a clinical point of view, identifying factors that influence pain perception is essential for improving patients’ experiences of dental care. Understanding how psychological states modulate pain [9] can help clinicians create environments that promote pain control, for example, by curating calm audiovisual content during procedures, especially for patients who fear pain or report dental anxiety. The findings from previous studies highlight the benefits of soothing office features, reading materials in waiting rooms, comfortable temperature, and music, which can reduce anxiety. Audiovisual distraction (e.g., 3D video glasses) has shown positive effects in anxious dental patients [55]. Also, pleasant ambient odours can lower anxiety and improve mood in the dental office [15]. Music therapy can reduce both pain and anxiety [56]. For highly anxious individuals, video and audio stimuli can be particularly useful to distract the patient during potentially painful procedures. In addition, evidence-based behavioural techniques, such as brief relaxation exercises, have been shown to successfully reduce dental anxiety [57].

Limitations. First, the pain stimulus used in this study was heat administered on the participants’ forearm, rather than an intra-oral pain stimulus. This raises the question as to how relevant these pain experiences and neural pathways are to the dental setting. Incorporating intra-oral pain stimuli to enhance clinical validity would be beneficial but would raise both methodological and ethical concerns. Second, the current experiment used a pain stimulus of a relatively short duration and fixed intensity, whereas experiences of real-world dental pain would probably vary in terms of intensity and duration. Finally, pain studies are susceptible to self-selection bias [58], given the focus on dentistry and pain, and participants willing to enrol may therefore differ from the broader patient population. The mean dental anxiety (MDAS) values reported in the current study appear somewhat lower than those reported elsewhere for adolescents [34], but comparable to those from a normal population [59]. The lack of blinding is a limitation of this study, and therefore performance bias cannot be excluded.

In conclusion, pain-relevant video stimuli were associated with higher pain ratings than non-pain-relevant video stimuli in a simulated dental setting. In contrast, neutral video and audio stimuli may have reduced pain ratings through attentional distraction. These findings suggest that attentional and emotional processes may influence pain perception during dental treatment.

## Figures and Tables

**Table 1:** Sample characteristics and baseline measures by group.

	Entire Sample (*n* = 80)	Video (*n* = 32)	Audio (*n* = 32)	Control (*n* = 16)
Female, *n* (%)	50 (62.50)	18 (56.30)	24 (75.00)	8 (50.00)
Age, M (SD)	22.66 (2.65)	22.50 (2.70)	22.63 (2.72)	23.06 (2.52)
MDAS, M (SD; CI)	8.55 (2.55; 7.97–9.13)	8.48 (2.71; 7.49–9.48)	8.68 (2.68; 7.69–9.66)	8.44 (2.06; 7.33–9.54)
SCL baseline (µS), M (SD; CI)	4.25 (2.23; 3.74–4.76)	4.20 (1.87; 3.51–4.89)	4.28 (2.13; 3.50–5.06)	4.31 (3.18; 2.47–6.15)
Pain baseline (0–100), M (SD; CI)	37.39 (23.63; 31.39–42.82)	37.65 (24.94; 28.50–46.79)	35.32 (20.14; 27.93–42.71)	41.69 (29.02; 24.16–59.23)
SACL baseline (0–10), M (SD; CI)	2.32 (1.99; 1.87–2.76)	2.16 (1.80; 1.50–2.80)	2.32 (1.99; 1.59–3.05)	2.63 (2.40; 1.34–3.91)

## Data Availability

The raw data supporting the conclusions of this article will be made available by the authors on request.

## Abbreviations

The following abbreviations are used in this manuscript:

CoVAS	Computerized Visual Analogue Scale
EDA	Electrodermal activity
MDAS	Modified Dental Anxiety Scale
SACL	Short Adjective Check List
SCL	Skin conductance level
VR	Virtual reality
